# Parents-child multiple sites of microbial and metabolic signatures in autism spectrum disorder

**DOI:** 10.3389/fmicb.2025.1745874

**Published:** 2026-01-22

**Authors:** Lingping Zhu, Haiyan Zhang, Meiling Tang, Xuefeng Yang, Yongjun Chen

**Affiliations:** 1 The Affiliated Nanhua Hospital, Department of General Practice, Hengyang Medical School, University of South China, Hengyang, Hunan, China; 2 School of Public Health, Fudan University, Shanghai, China; 3 The Affiliated Nanhua Hospital, Department of Pediatrics, Hengyang Medical School, University of South China, Hengyang, Hunan, China; 4 The Affiliated Nanhua Hospital, Department of Neurology, Hengyang Medical School, University of South China, Hengyang, Hunan, China

**Keywords:** autism spectrum disorder, dysbiosis, microbiota, oral-gut axis, parents-child

## Abstract

**Introduction:**

To investigate the horizontal transmission of oral-gut microbiota in autism spectrum disorder (ASD) families and its potential implications for ASD pathogenesis.

**Methods:**

The research employed a paired cohort design using family cohorts (23 ASD children/17 parents vs. 18 Non-ASD children/16 parents), conducting integrated microbiome and metabolomic analyses of oral and fecal samples.

**Results:**

The findings revealed that ASD families exhibited significantly increased oral microbial species diversity alongside substantial alterations in gut microbiota composition, particularly demonstrating a lower *Firmicutes*/*Bacteroidetes* ratio (3.60/2.97) compared to Non-ASD families (5.59/5.35). Specific microbial changes included notable enrichment of *Prevotella_9* in ASD gut microbiota. Metabolomic profiling identified significant disruptions in multiple metabolic pathways, including impaired L-rhamnose degradation and glutathione metabolism. The study observed coordinated oral-gut axis alterations through synchronized changes in *Caulobacter* and *Serratia* abundances, suggesting a distinct dysbiotic pattern along this microbial continuum. Additional metabolic findings demonstrated reduced levels of fecal glutamine and Ala-Gly in ASD children, with glycylproline exhibiting high predictive value for family typing (AUC = 0.91). Integrative analysis further revealed significant correlations between *Holdemanella* and various lipid metabolites.

**Discussion:**

It indicates that ASD families display characteristic oral-gut microbiota interactions accompanied by metabolic abnormalities, potentially reflecting familial microbial transmission patterns that may contribute to ASD pathophysiology.

## 1 Introduction

Autism spectrum disorder (ASD) is a neurodevelopmental disorder characterized by social communication difficulties, repetitive and stereotyped behaviors, and restricted interests, and is often accompanied by sensory abnormalities and communication issues (Li et al., 2024; Ahmadani et al., 2025). Its core features include deficits in social interactions (such as lack of eye contact and difficulty understanding others’ emotions) and restrictive/repetitive behavioral patterns (such as repetitive movements and intense fixation on specific objects) (Ahmadani et al., 2025). In recent years, the rate of ASD has increased significantly, with global data showing a rapid increase in prevalence, possibly due to updates in diagnostic criteria, environmental factors, and gene–environment interactions (Ahmadani et al., 2025; Li et al., 2023).

The transmission of symbiotic microorganisms from parents to children with ASD and how they affect the onset of the disease has not been studied in depth, but existing evidence suggests their potential importance. For example, abnormalities in the maternal gut microbiota may influence the offspring’s gut function and neurodevelopment through vertical transmission (Li et al., 2024). One study showed that maternal rats exposed to valproic acid (VPA) during pregnancy not only exhibited elevated levels of gut inflammatory factors and disrupted microbial community structure but also had offspring that displayed duodenal motility disorders and increased inflammatory factor levels (Li et al., 2024), suggesting that issues with maternal gut microbiota might impact the gut health and neurodevelopment of the offspring through the maternal–fetal axis. Additionally, microbial interventions (such as probiotic supplementation or fecal microbiota transplantation) might help improve behavioral abnormalities in ASD animal models. For instance, *Lactobacillus reuteri* can repair fear memory deficits in Cntnap4 gene-deficient mice by modulating GABAergic neurotransmission in the amygdala (Zhang et al., 2022), while the gut metabolite indole-3-propionic acid (IPA) improves social deficits in 16p11.2 microdeletion mice by activating the ERK1 pathway (Jiang et al., 2024). These results indirectly support the notion that the transmission of symbiotic microorganisms may influence ASD phenotypes.

It’s important to note that we have not directly studied the “horizontal transmission” of microorganisms between parents (such as microbial sharing between spouses or siblings). However, the composition of the gut microbiota is closely linked to the metabolism of neurotransmitters related to ASD (such as dopamine and GABA) (Gevi et al., 2020; Liu et al., 2022), and abnormalities in the metabolism of trace elements, such as zinc and vitamin B12, have also been associated with the dysregulation of the gut-brain axis in ASD (Ahmadani et al., 2025; Zwierz et al., 2025). Changes in these metabolic pathways may be influenced by the shared microbiota between the parents through interactions with the host. For example, elevated levels of 4-methylphenol in the urine of children with ASD may inhibit dopamine *β*-hydroxylase, affecting norepinephrine synthesis, and the production of this metabolite is related to the activity of specific gut microbiota (such as *Clostridium*) (Gevi et al., 2020).

Oral and gut microbiomes are intricately linked through the oral-gut axis. Microbes from the oral cavity, such as *Klebsiella* spp., can translocate to the gastrointestinal tract via swallowing, influencing gut microbial composition and function (Atarashi et al., 2017). This crosstalk can impact systemic health, including immune and metabolic responses. Dysbiosis in the oral microbiome (e.g., periodontitis) is associated with gut disorders like colorectal cancer, suggesting a bidirectional relationship mediated by microbial exchange, immune modulation, and metabolic interactions (Willis and Gabaldón, 2020).

Simultaneously, we should also examine the specific mechanisms of microbial transmission between parents, including the impact of maternal gut microbiota interventions during pregnancy on offspring neurodevelopment (Żebrowska et al., 2021; AlOlaby et al., 2022), as well as the possibility of paternal microbiota transmission through oral and skin contact.

To Identify gut microbial biomarkers for autism through comparisons with parents and healthy control families, we collected oral and fecal samples from families of Non-ASD children and families with children with autism. By examining the 16 s RNA of the oral and gut microbiota and gut metabolites, we aimed to provide evidence for studying the “horizontal transmission” of microorganisms between parents.

## 2 Methods

### 2.1 Experimental model and study participant details

This study included 23 children with autism spectrum disorder (ASD-C) and their parents (ASD-P), 18 neurotypical children (N-C), and 16 their parents (N-P). Both oral and fecal samples were collected. The inclusion criteria were as follows: (1) Children aged 3–15 years; adults aged 20–60 years; (2) no constipation or diarrhea in the past week, and (3) a diagnosis of autism confirmed by a certificate from a tertiary hospital or higher. Exclusion criteria: (1) Severe dysfunction of the heart, liver, kidneys, or other major organs; (2) Positive test for infectious disease pathogens; (3) Use of antibiotics, acid suppressants, or immunosuppressants in the past month; (4) Severe digestive system diseases, history of gastrointestinal surgery, or metabolic diseases; (5) History of malignant tumors; (6) Use of microecological regulators in the past month; (7) Pregnancy, breastfeeding, or severe malnutrition; (8) Coexisting neurological diseases such as epilepsy. The diagnosis of autism was confirmed by pediatricians. This study was approved by the Medical Ethics Committee of Nanhua Hospital, affiliated with University of South China (Approval No. 2024-KY-043). Samples were collected from March 2024 to November 2024, and the research protocol complied with the Declaration of Helsinki.

### 2.2 Sample collection methods

#### 2.2.1 Oral sample collection

Participants were instructed to fast for 1 h before sampling, only drink water, and not smoke or chew gum for at least 2 h. The mouth was rinsed with water 3 times (10 s each time) to remove food residue. The participant should sit up straight with their head slightly tilted, and adults should allow saliva to flow naturally into a centrifuge tube (avoiding active spitting), collecting a volume of ≥2 mL (at least 0.5 mL for DNA extraction). For children, a sterile cotton swab was used to wipe the submandibular/salivary gland area, and the swab was immersed in 1 mL sterile PBS and shaken for elution. The main collection time was between 9 and 11 a.m., and the samples were transferred to a −80 °C freezer within 30 min.

#### 2.2.2 Fecal sample collection

Antibiotics, probiotics, and laxatives were discontinued 48 h before sampling. Fresh feces were collected using sterile plastic containers, ensuring that they did not touch water or urine, and selecting the internal area of the feces (where the microbial distribution was more uniform). Samples (≥200 mg, approximately the size of a soybean) were processed within 15 min of exposure to air to minimize oxidative stress. The feces were placed in sterile tubes and transferred to a −80 °C freezer within 30 min.

### 2.3 Detection methods

#### 2.3.1 Fecal and saliva 16S RNA detection method

This process included five steps: extraction and detection of DNA, amplification with PCR, purification of the products, preparation of the library, and sequencing. First, fecal and saliva samples were separated, DNA was quantitatively analyzed using a Nanodrop instrument, and the quality of the extracted DNA was assessed using 1.2% agarose gel electrophoresis. To amplify the variable regions of the rRNA gene (single or continuous) or specific gene fragments, primers with sample-specific barcode sequences were designed based on the conserved regions of the sequence. The volume of PCR products is mixed at a ratio of 0.8 into 25 μL of PCR product and purified using Vazyme VAHTSTMDNA Clean Beads. Subsequently, quantitative analysis of the PCR amplification products was performed using fluorescence with a Quant-iT PicoGreen dsDNA detection kit and a microplate reader (BioTek, FLx800). Based on the fluorescence quantification results, each sample was mixed in appropriate proportions based on the required sequencing amount. An Illumina TruSeq Nano DNA LT library preparation kit was used to construct a sequencing library. The amplified products first repair the overhanging bases at the 5′ end of the DNA sequence by adding phosphate groups to complete the missing bases at the 3′ end, while adding an A base at the 3′ end to prevent self-aggregation of DNA fragments and ensure that the target sequence can attach to the sequencing adapter (i.e., the overhanging T base at the 3′ end). Additionally, sequencing adapters with library-specific tags (i.e., index sequences) are added to the 5′ end of the sequence to facilitate the fixation of DNA molecules on the flow cell. After the addition was complete, the library was purified using BECKMAN AMPure XP magnetic beads to remove self-associated fragments through bead selection and ligation. The ligated DNA fragments were subjected to PCR amplification to enrich the sequencing library template, and the enriched products were purified using BECKMAN AMPure XP magnetic beads. The final library was selected and purified by 2% agarose gel electrophoresis. Before sequencing, the quality of the library was tested using an Agilent Bioanalyzer and Agilent High Sensitivity DNA Kit. A qualified library exhibited a single peak without adapters. The library was quantified using the Promega QuantiFluor fluorescence quantification system and Quant-iT PicoGreen dsDNA detection kit with a qualified library concentration of 2 nM or above. The raw data obtained from high-throughput sequencing were first filtered based on sequence quality, and problematic samples were retested and replated. Using the analysis workflow of the QIIME2 dada2 or Vsearch software, the raw sequences that underwent preliminary quality screening were separated based on the index and barcode information, and barcode sequences were removed. The specific composition of each sample (group) at different classification levels is presented in an overview format. The *α* diversity level of each sample is assessed based on the distribution of ASV/OTU in different samples, while the appropriateness of sequencing depth is reflected by the rarefaction curve. At the ASV/OTU level, the distance matrix of each sample is calculated, and differences between different samples (groups) are measured through *β* diversity differences and their significance. Bray-Curtis distances were used to calculate β diversity, visualization tools such as PCOA to display sample clustering. Statistical tests like Adonis assess significance (*p*-value< 0.05 considered statistically significant) of group differences. Using various unsupervised ranking and clustering methods combined with appropriate statistical tests, further assessments of the differences in species abundance between samples (groups) were conducted. At the species classification composition level, attempts have been made to identify marker species using various unsupervised and supervised ranking, clustering, and modeling tools, along with corresponding statistical tests. Based on the distribution of species in the samples, association networks were constructed, topological indices were calculated, and key species were identified. Furthermore, based on the sequencing results of the 16S rRNA, 18S rRNA, and ITS genes, the metabolic functions of the sample communities can also be predicted, identifying differential pathways and obtaining the species composition of specific pathways. The bioinformatics analysis is referenced from previous literature (Zhou et al., 2022).

#### 2.3.2 Fecal LC-MS metabolite analysis

This divided into three steps: metabolite extraction, on-machine detection, and off-machine data processing. A 25 mg portion of each sample was weighed at low temperature and transferred to an EP tube containing two homogenization beads. Subsequently, 500 μL of extraction solution [methanol: acetonitrile: water = 2:2:1 (V/V)], containing isotopically labeled internal standards was added. This mixture was vortexed for 30 s, homogenized at 35 Hz for 4 min, then transferred to an ice-water bath for ultrasonic treatment for 5 min. This step was repeated 3 times. The samples were then incubated at −40 °C for 1 h and centrifuged at 4 °C, 12000 rpm (centrifugal force 13,800 (×*g*), radius 8.6 cm) for 15 min. The supernatant was taken for injection into the detection machine and equal amount of supernatant from all samples was mixed to form a QC sample for detection. For polar metabolites, Vanquish (Thermo Fisher Scientific) ultra-high performance liquid chromatography system was used, with Waters ACQUITY UPLC BEH Amide (2.1 mm × 50 mm, 1.7 μm) liquid chromatography column for chromatographic separation of target compounds. Mobile phase A was an aqueous solution containing 25 mmol/L ammonium acetate and 25 mmol/L ammonia, whereas phase B was acetonitrile. Sample tray temperature was 4 °C and injection volume was 2 μL. The Orbitrap Exploris 120 mass spectrometer performed the first and second mass spectrometry data acquisition under the control of the software (Xcalibur, version 4.4, Thermo). The instrument settings were as follows: Sheath gas flow rate: 50 Arb, Aux gas flow rate: 15 Arb, Capillary temperature: 320 °C, Full ms resolution: 60000, MS/MS resolution: 15000, Collision energy: SNCE 20/30/40, Spray Voltage: 3.8 kV (positive) or −3.4 kV (negative). The raw data were converted to mzXML format using ProteoWizard software, and metabolite identification was performed using a collaboratively developed R package with the database BiotreeDB (V3.0), followed by visualization analysis using a self-developed R package. The raw data included 9 quality control (QC) samples and 80 experimental samples, from which 39,823 features were extracted. The model construction process employed principal component analysis (PCA), orthogonal partial least squares discriminant analysis (OPLS-DA), and partial least squares (PLS) methods. The metabolomic profile is displayed as a score plot, with each data point representing a sample. Corresponding loading plots and S-plots were generated to provide information on the metabolites affecting sample clustering. OPLS-DA identifies key metabolites through variable importance projection (VIP). To identify variables that significantly contributed to the classification, *p*-values, VIP values generated by OPLS-DA, and fold changes (FC) were used. Ultimately, variables with p-values less than 0.05 and VIP values greater than 1 were considered significant. Metabolites identified through metabolomics were mapped to KEGG pathways for higher-level systematic functional biological interpretations. Additionally, the relationship between metabolomics and gut microbiota was analyzed using Spearman’s correlation coefficients, and potential biomarkers were explored using ROC curves.

### 2.4 Statistical method

Group Comparison and Statistical Methods: To explore the relationships between the groups, our comparative measurements included comparisons between ASD-C and ASD-P, ASD-C and N-C, and ASD-P and N-P groups, while comparing the shared microbiota and metabolites of families with autism with those of Non-ASD families to study intergenerational transmission. Statistical analyses were performed using the IBM SPSS Statistics software (version 25.0). Descriptive data were analyzed using *t*-tests, with *p* < 0.05 considered statistically significant, and missing values were replaced with the mean.

Bioinformatic analyses were performed in R (version 4.4.2), The Benjamini-Hochberg multiple comparison correction method was used to control the false discovery rate (FDR) regard with *α* diversity, with statistical significance defined as a FDR-adjusted q value < 0.05. The Kruskal-Wallis test was used to assess inter-group differences, followed by pairwise Dunn’s test with FDR correction; PERMANOVA analysis was performed within the PERMANCOVA framework (adonis2), including age as a covariate regard with *β* diversity. The non-parametric Kruskal-Wallis test (to assess overall group effects) was used to analyze the differences in relative abundance of bacterial phyla and genera among groups, followed by pairwise Wilcoxon rank-sum tests and FDR correction. Logarithmic fold change (log₂FC) was calculated based on the median abundance; KEGG pathway differential abundance: The DESeq2 package (negative binomial Wald test) was used to analyze the differential abundance of KEGG pathways predicted by PICRUSt2, which can handle compositional and variance issues, and perform FDR correction.

## 3 Results

### 3.1 Basic information

The ASD-C group included 23 cases, with an average age of 6.86 ± 2.95, comprising 3 girls and 20 boys. The N-C group included 18 cases, with an average age of 6.94 ± 2.53, including 12 girls and 6 boys. There was no statistically significant difference in age between the two groups (*p* > 0.05). The ASD-P group had 17 cases, consisting of 15 mothers and 2 fathers, with an average age of 41.87 ± 4.39. The N-P group had 16 cases, all mothers, with an average age of 37.75 ± 3.34, showing a statistical difference in age (*p* = 0.006).

### 3.2 Analysis of microbiome 16s RNA sequencing

#### 3.2.1 Analysis of oral microbiome 16s RNA sequencing

**Figure 1:**
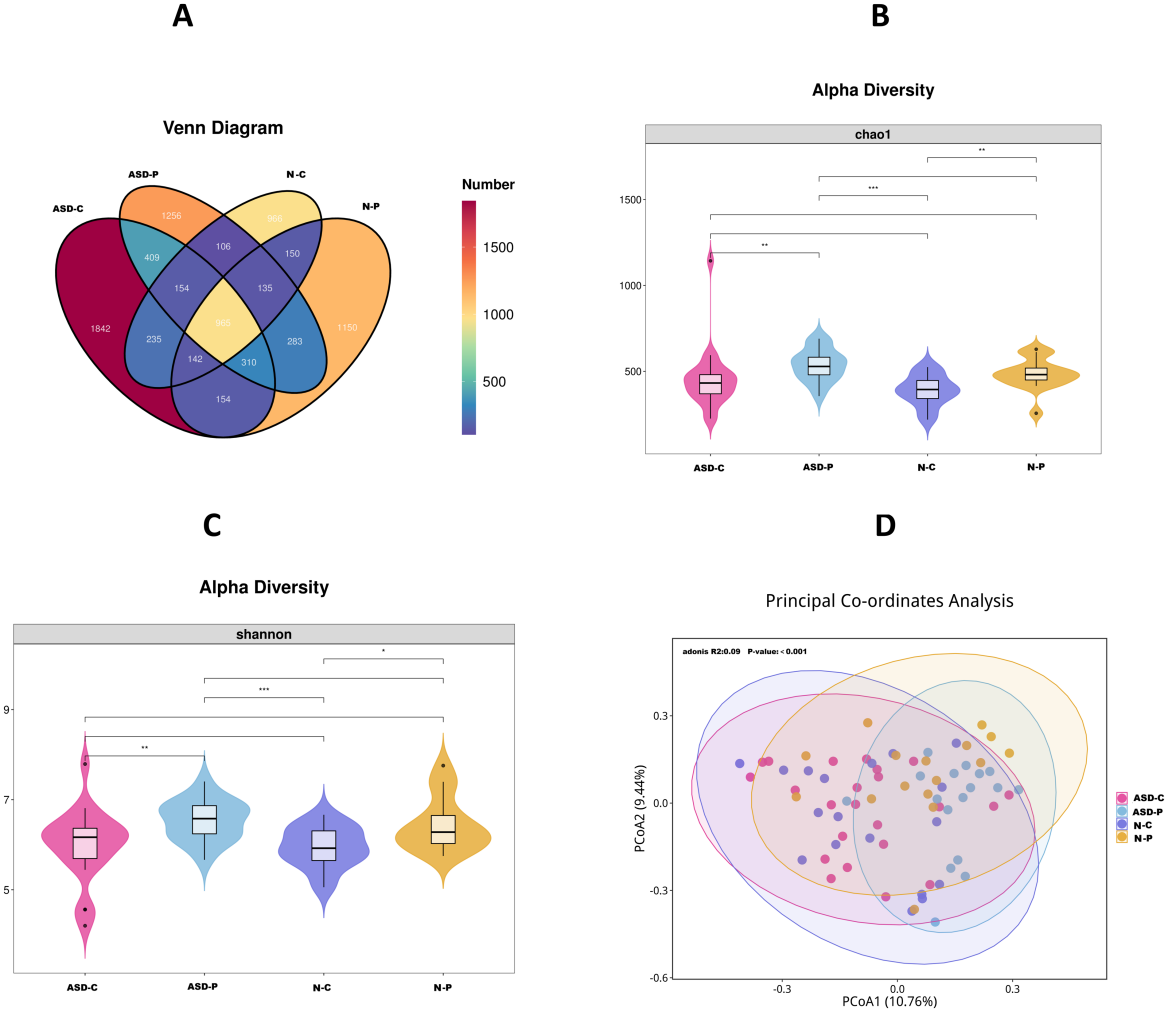
Comparison of oral microbiome 16S RNA sequencing data. ASD-C, autism child; ASD-P, family members of autism child; N-C, non-ASD child; N-P, family members of non-ASD child. **(A)** Venn diagram of oral microbial species distribution in each group. **(B)** Chao1 index of alpha diversity between four groups. **(C)** Shannon index of alpha diversity between four groups. **(D)** Principal coordinates analysis of groups between four groups.

Analysis of the oral microbiome 16 s RNA results showed that the ASD-C and ASD-P groups shared 1,838 species of oral microbiomes, whereas the N-C and N-P groups shared 1,392 species. The number of common oral microbial species was 1,496 between the ASD-C and N-C groups, and 1,693 between the ASD-P and N-P groups. The total number of oral microbiome species in the ASD-C and ASD-P groups was significantly higher than that in the N-C and N-P groups (Figure 1A). There were significant differences in chao1 Alpha diversity among the ASD-C, ASD-P, N-C, and N-P groups (Figure 1B), and similar results were observed for Shannon alpha diversity (Figure 1C). Principal coordinate analysis clearly distinguished families with autism from Non-ASD families (Figure 1D). At the Phylum level, the main groups were *Firmicutes*, *Proteobacteria*, *Bacteroidetes*, *Actinobacteria*, *Spirochaetota*, *Campylobacterota*, and *Fusobacteriota*, with the most abundant genus being *Firmicutes*. The top five genera were *Streptococcus*, *Veillonella*, *Neisseria*, *Haemophilus*, and *Prevotella_7* (Supplementary Figures S1A,B). Correlation analysis of the 30 most abundant bacteria revealed that *Streptococcus* was positively correlated with *Granulicatella* and *Gemella* and negatively correlated with *Prevotella*, *Campylobacter*, *Treponema*, and *Fusobacterium*. *Veillonella* was positively correlated with *Megasphaera*, *Prevotella_7*, and *Actinomyces*, while *Prevotella_7* was positively correlated with *Megasphaera* and *Prevotella* and negatively correlated with *Lautropia* (Supplementary Figure S1C). METACYC functional analysis of the oral microbiomes in the ASD-C and ASD-P groups revealed significant differences in aerobactin biosynthesis, chondroitin sulfate degradation I (bacterial), colanic acid building block biosynthesis, GDP-mannose biosynthesis, heme biosynthesis II (anaerobic), and L-arginine biosynthesis I (via L-ornithine) (Supplementary Figure S1D).

#### 3.2.2 Analysis of fecal microbiome 16s RNA sequencing

**Figure 2:**
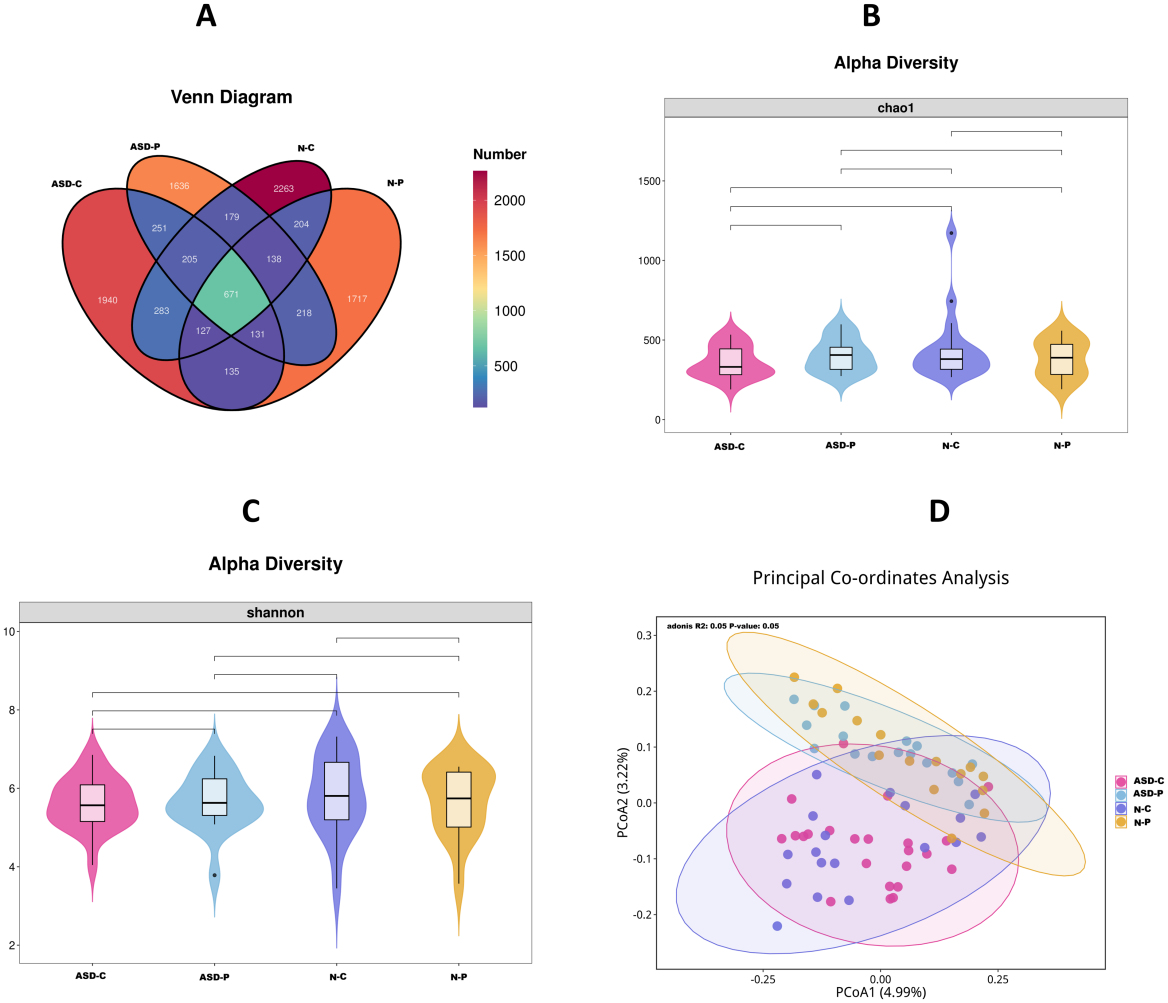
16S RNA sequencing data comparison of fecal microbiota. ASD-C, Autism child; ASD-P, family members of autism child; N-C, non-ASD child; N-P, family members of non-ASD child. **(A)** Top 30 relative abundance of genus between four groups. **(B)** Sankey plots of genus abundance between four groups. **(C)** Genus correlation heatmap of top 30 abundance between four groups. **(D)** Different function prediction with MetaCyc database between ASD-C and ASD-P group.

Analysis of the fecal microbiome 16s RNA results showed that the ASD-C and ASD-P shared 1,258 species of fecal microbiomes, whereas the N-C and N-P shared 1,140 species. The number of common fecal microbial species was 1,286 between the ASD-C and N-C groups, and 1,158 between the ASD-P and N-P groups. Non-ASD children had a greater variety of gut microbiome species than children with autism (Figure 2A). There were no significant differences in Shannon and Chao1 alpha diversity among the groups (Figures 2B,C). After principal coordinate analysis, the children’s group was clearly distinguished from the adult group (Figure 2D). At the phylum level, the main groups included *Actinobacteriota*, *Bacteroidota*, *Firmicutes*, *Proteobacteria*, and *Verrucomicrobiota*, with most genera belonging to *Firmicutes*. The *Firmicutes*/*Bacteroids* ratios for ASD-C, ASD-P, N-C, and N-P were 3.60, 2.97, 5.59, and 5.35, respectively. The top five genera were *Faecalibacterium*, *Prevotella_9*, *Bacteroides*, *Megamonas*, and *Bifidobacterium* (Supplementary Figure S2A). Notably, *Prevotella_9* was significantly abundant in families with autism than in Non-ASD families, whereas Bifidobacterium was most abundant in the N-C group (Supplementary Figure S2B). Correlation analysis of the top 30 differential bacteria showed that *Bacteroides* was positively correlated with *Ruminococcus gnavus*, which was negatively correlated with *Coprococcus*, *Christensenellaceae_R-7*, and UCG-002 (Supplementary Figure S2C). METACYC functional analysis of fecal microbiomes in the ASD-C and ASD-P groups revealed significant differences in L-rhamnose degradation II, L-rhamnose degradation III, sucrose biosynthesis I (from photosynthesis), and sucrose biosynthesis III (Supplementary Figure S2D).

#### 3.2.3 Differences and relations between oral and fecal microbiome 16s RNA sequencing

Differences in *Streptococcus* and *Prevotella* were observed in both oral and fecal samples, with *Streptococcus* being the most abundant in both oral and fecal samples of N-C, whereas *Prevotella* was the least abundant in fecal samples. Interestingly, when comparing the oral and fecal microbiomes of the ASD-C and N-C groups, we found that *Neisseria*, *Methyloversatilis*, *Paracoccus*, *Caulobacter*, *Neisseriaceae_unclassified*, *Anaerotruncus*, and *Serratia* were different at both sites, with consistent changes observed in *Caulobacter* and *Serratia*.

### 3.3 Analysis of fecal metabolites LC-MS

**Figure 3:**
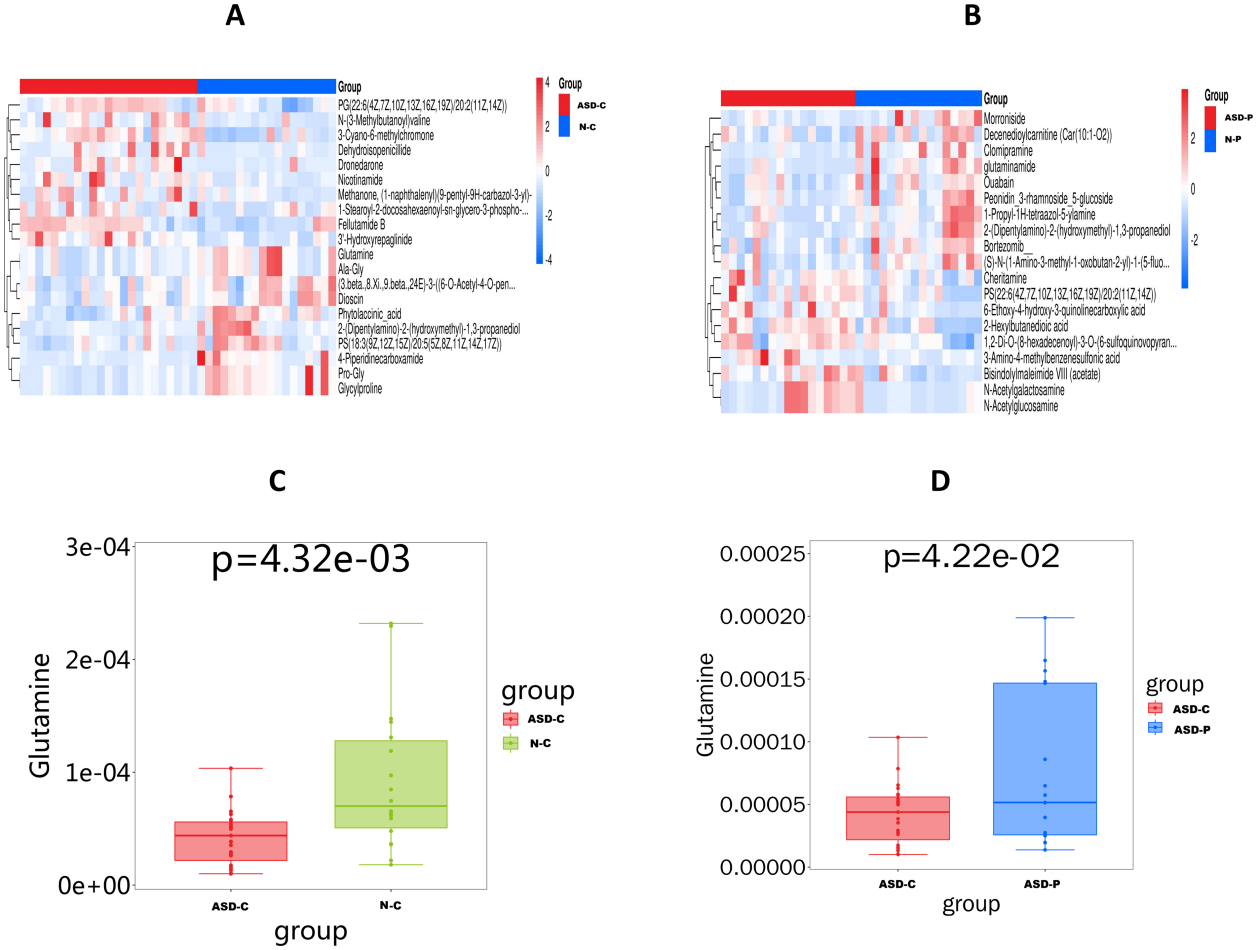
Analysis results of differential metabolites in feces. ASD-C, autism child; ASD-P, family members of Autism child; N-C, non-ASD child; N-P, family members of non-ASD child. **(A)** Heatmap of the top 10 significantly upregulated and downregulated differential substances (ASD-C vs. N-C). **(B)** Heatmap of the top 10 significantly upregulated and downregulated differential substances (ASD-P vs. N-P). **(C)** Bar chart of fecal glutamine content differences between ASD-C and N-C. **(D)** Bar chart of fecal glutamine content differences between ASD-P and N-P.

**Figure 4:**
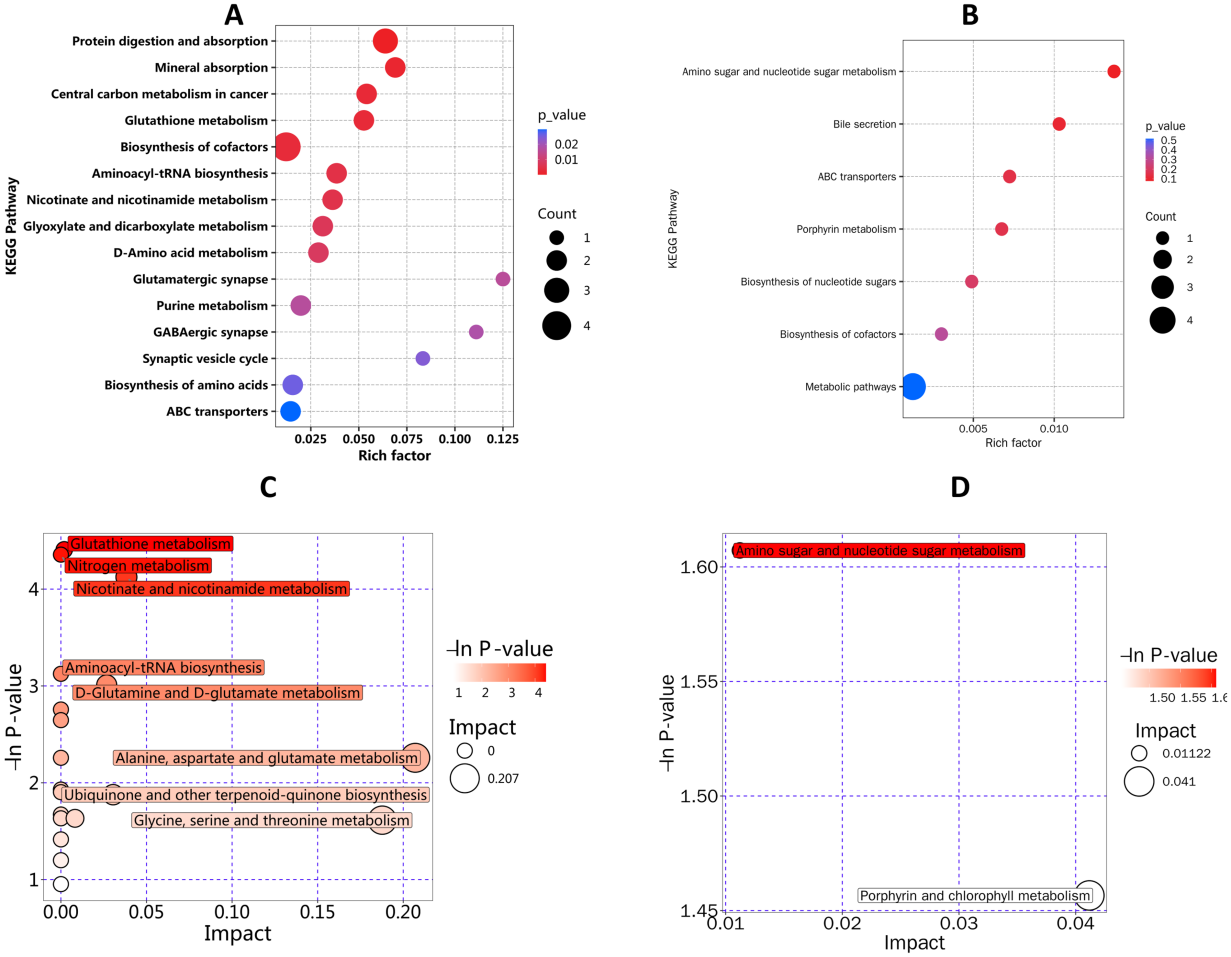
Differential metabolite pathway prediction. ASD-C, autism child; ASD-P, family members of autism child; N-C, non-ASD child; N-P, family members of non-ASD child. **(A)** KEGG enrichment map of differential metabolites in the ASD-C group compared to the N-C group. **(B)** KEGG enrichment map of differential metabolites in the ASD-P group compared to the N-P group. **(C)** Pathway analysis map of differential metabolites in the ASD-C group compared to the N-C group. **(D)** Pathway analysis map of differential metabolites in the ASD-P group compared to the N-P group.

Analysis of the fecal metabolites results revealed significant differences in metabolites between ASD-C and N-C, including Glutamine, Ala-Gly, Fellutamide B, Nicotinamide, and 3’-Hydroxyrepaglinide (Figure 3A). Significant differential metabolites between the ASD-C and N-P groups included morroniside, ouabain, clomipramine, N-acetylgalactosamine, glutaminamide, and 3 − Amino−4−methylbenzenesulfonic acid (Figure 3B). Additionally, Glutamine and Ala-Gly levels were lower in the ASD-C group than in the ASD-P and N-C groups (Figures 3C,D and Supplementary Figures S3A,B). Twenty differential metabolites were identified in families with autism and 22 in families without autism (Supplementary Figure S3C). We performed *K*-means clustering analysis on all differential metabolites, resulting in nine groups, with 110 metabolites being significantly lower in families with autism than in Non-ASD families (Supplementary Figure S3D). KEGG analysis of the differential metabolites between children with autism and Non-ASD children revealed differences in protein digestion and absorption, mineral absorption, central carbon metabolism in cancer, glutathione metabolism, and cofactor biosynthesis (Figure 4A). The differential metabolite KEGG functional differences between parents with autism and Non-ASD parents were mainly associated with amino sugar and nucleotide sugar metabolism, bile secretion, ABC transporters, biosynthesis of nucleotide sugars, biosynthesis of cofactors, and metabolic pathways (Figure 4B). The differential metabolic pathways between children with autism and Non-ASD children mainly differed in glutathione metabolism, nitrogen metabolism, nicotinate and nicotinamide metabolism, and aminoacyl tRNA biosynthesis (Figure 4C). The differential metabolite pathways between parents with autism and Non-ASD parents mainly differed in amino sugar and nucleotide sugar metabolism, and porphyrin and chlorophyll metabolism (Figure 4D). Additionally, we found that Ala-Gly and 2-(dipentylamino)-2-(hydroxymethyl)-1,3-propanediol had predictive values across the different groups (Supplementary Figure S4).

### 3.4 Comparison of oral microbiomes between autism families and non-ASD families

Comparison revealed that the Alpha Diversity of oral microbiomes was slightly higher in families with autism than in Non-ASD families (Supplementary Figure S5A), but the difference was not statistically significant. At the genus level, the main groups included *Firmicutes*, *Proteobacteria*, *Bacteroidota*, *Fusobacteriota*, *Actinobacteriota*, *Campylobacterota*, and *Spirochaetota*, with most of the genera found in *Firmicutes* (Supplementary Figure S5B). *Streptococcus*, *Veillonella*, *Neisseria*, *Haemophilus*, and *Prevotella_7* occupied the top five positions at the species level. Notably, the oral microbiome in families with autism showed significant increases in *Neisseria*, *Eikenella*, *F033*2, *Enhydrobacter*, and *Deinococcus*, whereas *Bergeyella*, *Caulobacter*, and *Methyloversatilis* showed significant decreases (Supplementary Figure S5C). Correlation analysis of the 30 most abundant bacteria revealed that *Streptococcus* was positively correlated with *Granulicatella* and *Gemella* and negatively correlated with *Prevotella*, *Treponema*, and *Campylobacter*. *Actinobacillus* was significantly positively correlated with *Pseudomonas*, *Fusobacterium* was significantly positively correlated with *Porphyromonas*, and *Megasphaera* was significantly positively correlated with *Prevotella_7* (Supplementary Figure S5D).

### 3.5 Comparison of fecal microbiomes between autism families and non-ASD families

**Figure 5:**
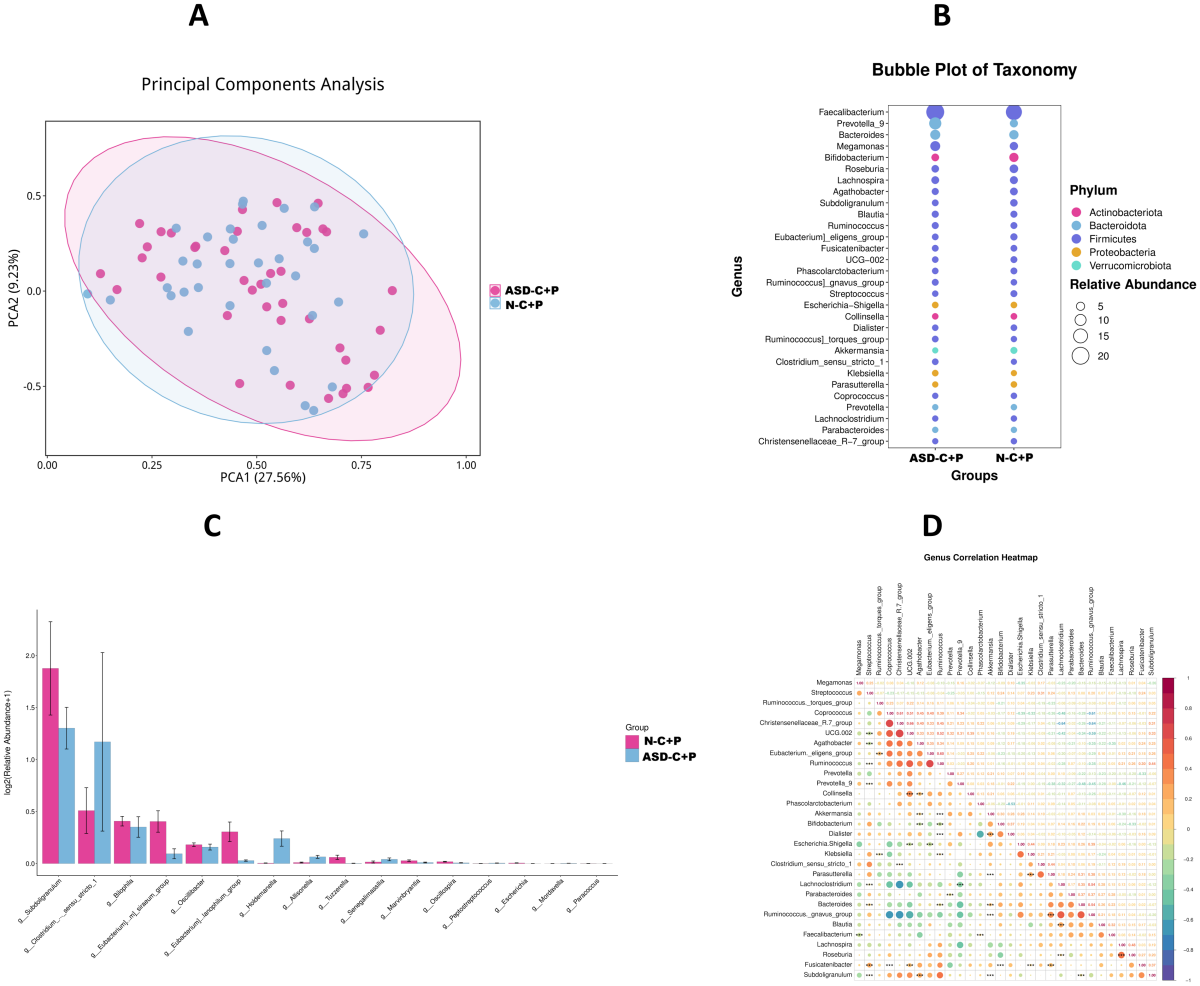
Comparison of fecal microbiota 16S RNA between autism spectrum disorder families and non-ASD families. ASD-C + P, autism spectrum disorder families; N-C + P, non-ASD families. **(A)** Principal components analysis of fecal microbiota between ASD-C + P and N-C + P. **(B)** Bubble plot comparing the top 30 abundance of fecal microbiota at the phylum level between ASD-C + P and N-C + P. **(C)** Barplot comparing the abundance of fecal microbiota at the genus level between ASD-C + P and N-C + P. **(D)** Fecal genus correlation heatmap of differential abundance between ASD-C + P and N-C + P.

There was no significant difference in the beta diversity of the fecal microbiomes between the two families (Figure 5A). The phylum-level composition of fecal microbiomes in both families mainly included *Firmicutes*, *Bacteroidota*, *Actinobacteriota*, *Proteobacteria*, and *Verrucomicrobiota*, with most genera found in *Firmicutes* (Figure 5B). In families with autism, the abundance of *Clostridiumsensustricto1*, *Holdemanella*, *Allisonella*, and *Senegalimassilia* significantly increased, whereas there was a significant decrease in the abundance of *Subdoligranulum*, *Bilophila*, *Eubacterium siraeum*, *Oscillibacter*, *Eubacterium xylanophilum*, and *Tuzzerella* (Figure 5C). Correlation analysis of the 30 most abundant bacteria revealed that *Ruminococcus gnavus* was positively correlated with *Bacteroides*, and *Eubacterium eligens* and *UCG.002* were significantly positively correlated with *Ruminococcus*, whereas *Christensenellaceae_R-7* was significantly positively correlated with *Coprococcus* and *UCG.002* (Figure 5D). Differences in the oral and fecal microbiomes included *Paracoccus* and *Eubacterium xylanophilum*, although these changes were inconsistent.

### 3.6 Comparison of differential fecal metabolites between autism families and non-ASD families

**Figure 6:**
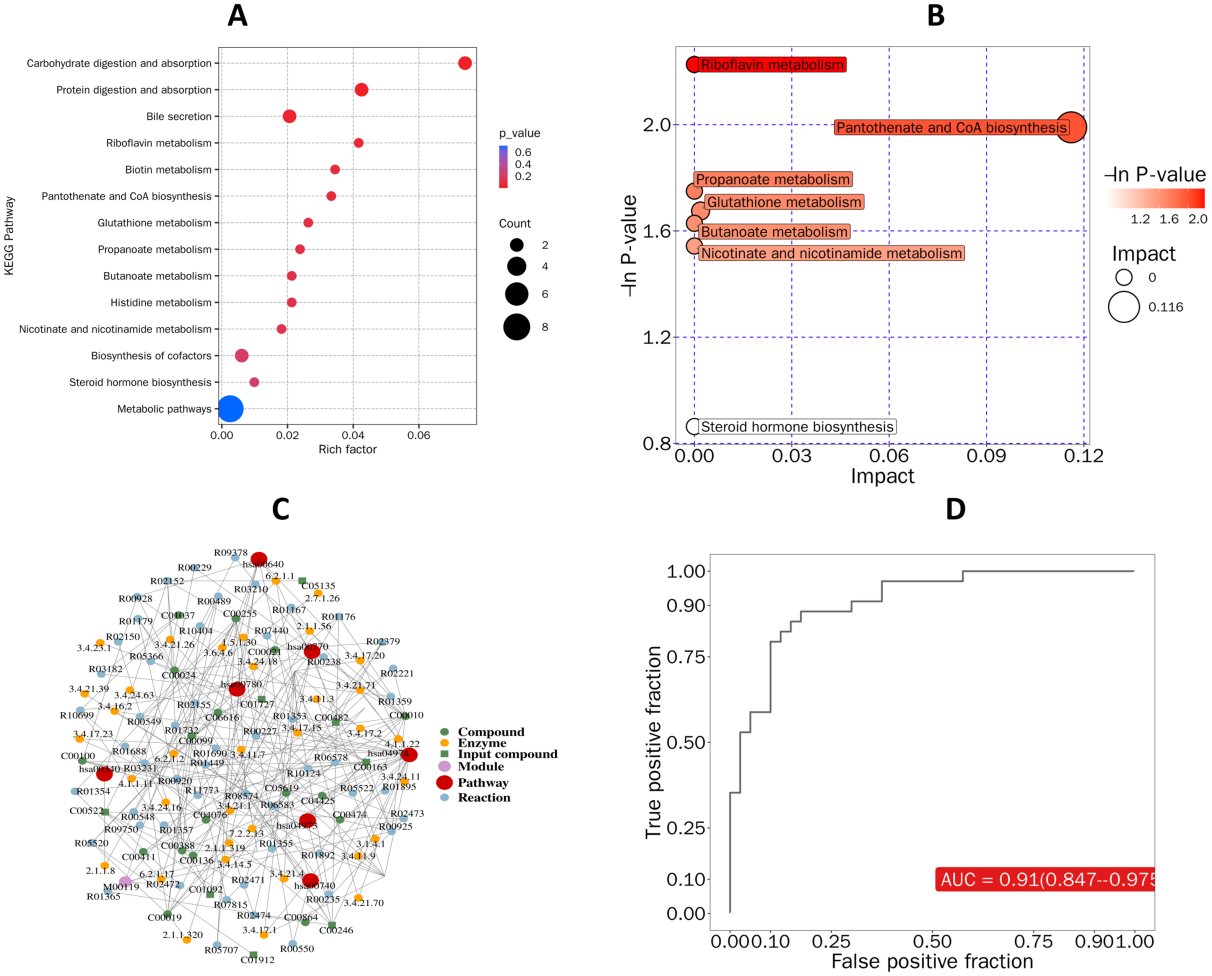
Prediction of functional differences in fecal metabolites. ASD-C + P, autism spectrum disorder families; N-C + P, non-ASD families. **(A)** KEGG pathway prediction of differential fecal metabolites between ASD-C + P and N-C + P. **(B)** Pathway analysis bubble plot of differential fecal metabolites between ASD-C + P and N-C + P. **(C)** Differential metabolite regulatory network analysis diagram. **(D)** ROC analysis diagram of Glycylproline to distinguish two families.

Supplementary Figure S6A shows 10 upregulated and 10 downregulated metabolites in the two families, with notably decreased levels of metabolites in families with autism including glutaminamide, Pro-Gly, Glycylproline, Phytolaccinic_acid, 2-(Dipentylamino)-2-(hydroxymethyl)-1,3-propanediol, and Nivalenol (Supplementary Figure S6B). Metabolites with significantly increased levels included fellutamide B, 2−hexylbutanedioic acid, 3′−hydroxyrepaglinide, 3−cyano−6−methylchromone, indole−3−propionic acid, and indole−3−methyl acetate (Supplementary Figure S6C). Metabolites that were positively correlated with glutaminamide included calealactone B, nivalenol, 2−(dipentylamino)−2−(hydroxymethyl)−1,3−propanediol, M408T172, 1−propyl−1H−tetraazol−5−ylamine, Pro-Gly, and Glycylprolin, while those negatively correlated included 2−Hexylbutanedioic acid, Fellutamide B, and Propionic acid (Supplementary Figure S6D). KEGG functional differences included metabolic pathways, protein digestion and absorption, and cofactor biosynthesis (Figure 6A), with pathway differences mainly in riboflavin metabolism, Pantothenate and CoA biosynthesis, propanoate metabolism, glutathione metabolism, butanoate metabolism, nicotinate and nicotinamide metabolism, and steroid hormone biosynthesis (Figure 6B). Figure 6C shows the regulatory network analysis of differential metabolites in feces, and Figure 6D shows the predictive value of glycylproline in the two families, with an AUC of 0.91 (0.847–0.975), significantly distinguishing the two families.

### 3.7 Correlation analysis of fecal microbiomes and metabolites between autism families and non-ASD families

**Figure 7:**
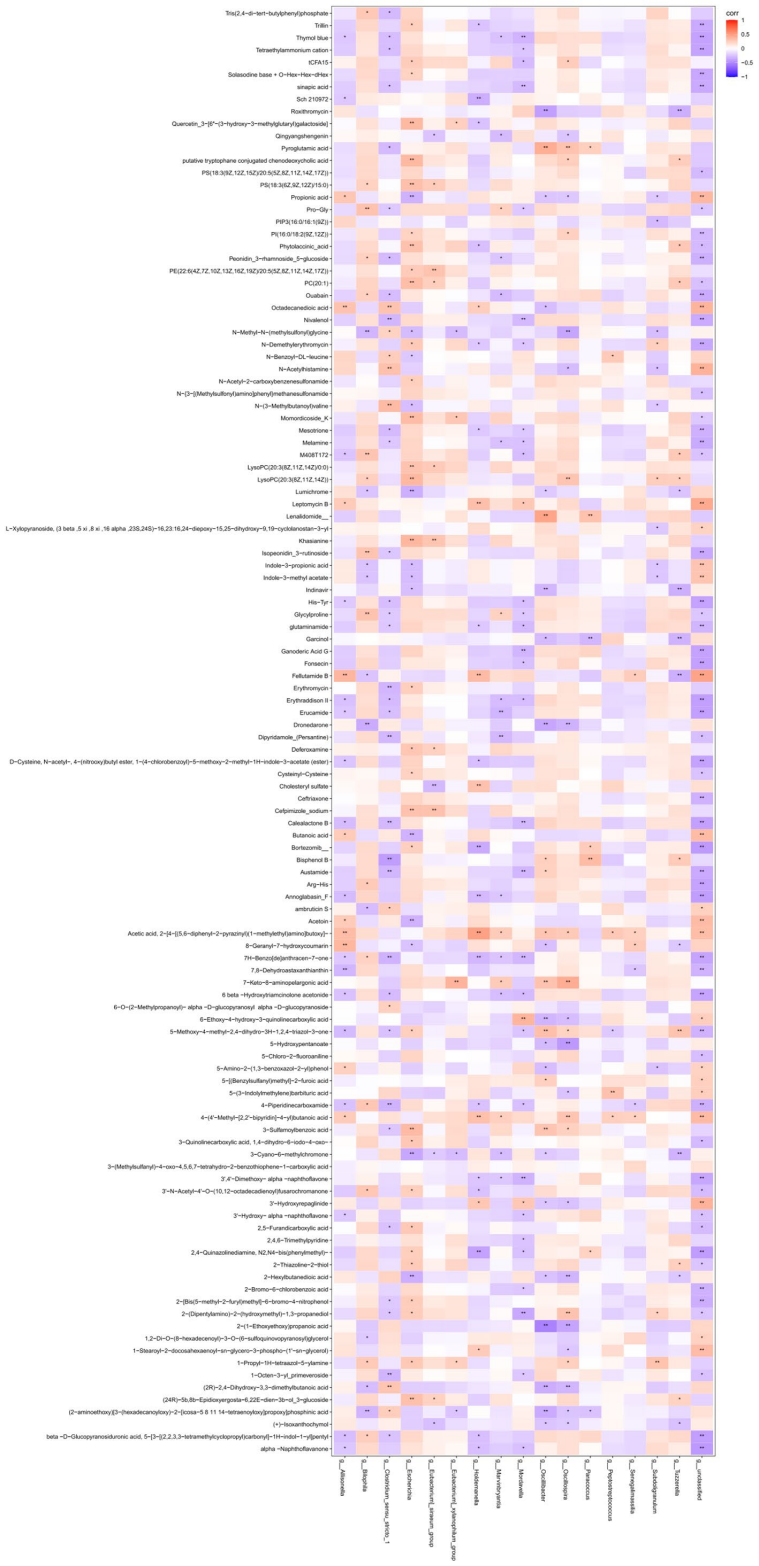
Correlation analysis heatmap of fecal microbiota and metabolic products differences between autism families and non-ASD families. * means *p* value<0.05, ** means *p* value<0.01.

The correlation analysis of gut microbiomes and metabolites between the two families showed that *Holdemanella* was linked to Sch 210972, Leptomycin B, 7H-benzo[de]anthracen-7-one, bortezomib, 2,4-quinazolinediamine, N2, N4-bis(phenylmethyl), cholesteryl sulfate, 4-(4′-methyl-[2,2′-bipyridin]-4-yl)butanoic acid, leptomycin B, fellutamide B, acetic acid, and 2-[4-[(5,6-diphenyl-2-pyrazinyl)(1-methylethyl)amino]butoxy]- (Figure 7).

## 4 Discussion

This study is the first to explore key differences in oral and gut microbiomes and metabolomes between families of children with autism spectrum disorder (ASD) and Non-ASD controls using a multi-site and multi-omics approach. This study found that oral microbial diversity in ASD families was notably increased, characterized by an increased abundance of *Neisseria* and a decreased abundance of *Caulobacter*. The oral cavity hosts a complex and diverse microbial community composed of various microbial species, including bacteria, viruses, fungi, and archaea, which helps maintain oral health by interacting with the host immune system (Griffen et al., 2011; Morrison et al., 2023). Previous studies have indicated that the oral microbiome may contribute to various neurological diseases such as Alzheimer’s disease, seizures, multiple sclerosis, migraines, and Parkinson’s disease (Olsen and Hicks, 2019). Earlier research has shown that the oral microbial composition of children with ASD differs from that of neurotypical (NT) siblings, identifying 108 differential species (*q* < 0.005) (Manghi et al., 2024); however, this study did not specifically mention the trends of *Neisseria* or *Caulobacter*. Additionally, a meta-analysis suggested that Rothia is enriched in the oral cavity of children with ASD, but does not involve the two aforementioned genera (Cao et al., 2025). Previous studies have found an increased abundance of *Porphyromonas* and *Prevotella* species in the oral cavity of children with ASD, which are typically associated with oral infections, such as gingivitis and periodontitis, indicating a potential link between oral inflammation and wider ASD symptoms (Kunath et al., 2024). *Neisseria* is generally presented as an opportunistic pathogen, leading to various diseases, including severe manifestations, such as endocarditis, with a weakened immune system being the main risk factor for infection (Walsh et al., 2023). Further investigations are required to determine whether this increases the risk of ASD. *Caulobacter* is currently considered a non-pathogenic microorganism (Iyer-Biswas et al., 2014), and its role as a protective factor for ASD has not been proven. The increased oral microbial diversity might indicate the influence of ASD-related immune or environmental factors.

The present study found that in the gut microbiome of children with ASD, *Prevotella_9* was elevated, whereas *Bifidobacterium* was decreased. *Prevotella_9* was also elevated in the feces of their parents. Interestingly, a previous study involving 40 patients found that *Prevotella_9* was significantly elevated in children with ASD and constipation (He et al., 2023), which aligns with the results of the present study. Previous research has shown that amino acids, carbohydrates, and lipids associated with the gut-brain axis in ASD are linked to gut microbes such as *Prevotella* and *Bifidobacterium* (Morton et al., 2023). Studies have indicated that an increase in *Prevotella* correlates with certain symptoms of ASD, such as social deficits and behavioral issues (Wan et al., 2022). Previous studies have found that certain harmful bacteria (e.g., *Klebsiella*) are significantly increased in the gut microbiome of patients with ASD, while beneficial bacteria (e.g., *Bifidobacterium*) are significantly decreased (De Sales-Millán et al., 2023). Children with ASD had significantly lower levels of *Bifidobacterium* in their feces than Non-ASD children, which correlated with the severity of gastrointestinal symptoms (Tomova et al., 2015). This phenomenon has been confirmed by previous studies, suggesting that the reduction of *Bifidobacterium* could worsen gut-brain axis dysfunction by affecting bile acid metabolism (e.g., inhibiting the proliferation of *Desulfovibrio*) and tryptophan metabolic pathways (Golubeva et al., 2017). After administering a probiotic combination containing *Bifidobacterium longum*, the levels of *Bifidobacterium* in the feces of children with ASD increased significantly, and gastrointestinal symptoms (such as constipation and diarrhea) and behavioral symptoms (ABC scale scores) improved (Shaaban et al., 2018). This study also showed that the levels of *Bifidobacterium* in the guts of parents of children with autism did not significantly decrease, indicating that it may only affect the development of ASD at certain ages. The increase in *Prevotella_9* and decrease in *Bifidobacterium* might disrupt short-chain fatty acid metabolism, thereby affecting the gut-brain axis.

At the same time, we also found that the fecal metabolites glutamine and Ala-Gly in children with autism were significantly reduced. Glutamine is a key energy source and neurotransmitter precursor (such as glutamate and GABA) for the central nervous system, and a decrease in glutamine level could increase oxidative stress and neuroinflammation through abnormal glutathione metabolic pathways, participating in the pathophysiology of ASD. Studies have shown that, compared to the control group, children with ASD exhibit significantly elevated plasma levels of GABA and a lower ratio of glutamate/glutamine, as well as significantly lower levels of plasma glutamine and glutamate/GABA ratio (Al-Otaish et al., 2018). Although previous studies have primarily used plasma glutamine for research, the levels of glutamine in feces corroborate previous findings. However, the relationship between fecal Ala-Gly and autism has not been documented, although metabolomic analyses have revealed abnormalities in various metabolites in the urine of ASD children (Gevi et al., 2020). We also found that glycylproline showed promise for the early identification of high-risk ASD families (AUC = 0.91), and various gut metabolites in families with autism exhibited consistent trends, indicating its potential for early identification of high-risk ASD families, which requires further exploration in the future.

This study is the first to discover synergistic dysbiosis of *Serratia* and *Caulobacter* in the oral cavity and gut of children with ASD through a cross-area analysis, a phenomenon that has not been reported in previous studies. Oral microbes can migrate to and colonize the gut. The microbial community on the tongues of children with ASD (such as *Haemophilus*) is significantly correlated with the gut microbiota, and joint analysis can predict the efficacy of fecal microbiota transplantation (WMT) in children with ASD (Zhong et al., 2025), suggesting that oral microbes might be closely linked to the gut microbiota. Some studies have suggested that oral microbes can move to the gut, affecting the gut-brain axis and linking it to the onset of ASD (Wan et al., 2024). The results of this study indicate that the synergistic dysbiosis of oral and gut bacteria might partially support this theory. However, other studies have shown a significant correlation between ASD, feeding disorders, and changes in the gut microbiota (D'Angelo et al., 2025), which might also mean that dietary restrictions shape the oral-gut axis in children with autism.

This study showed that, although there was no significant difference in the types and abundance of gut microbiota between parents of children with autism and parents of Non-ASD children, the gut B/F ratio of parents of children with autism was significantly lower, showing some level of gut microbiota imbalance. The gut microbiota is unique to each individual and begins to form at birth. This ecosystem is shaped by factors such as prematurity, how the baby was born, and breastfeeding (Caprara et al., 2024). Previous studies have shown that pregnant women experience dysbiosis of the maternal gut microbiota, leading to changes in the gut microbiota of their kids (Nyangahu and Jaspan, 2019). Mothers of children with autism reportedly have higher abundances of *Proteobacteria*, *Alphaproteobacteria*, *Moraxellaceae*, and *Acinetobacter* than mothers of Non-ASD children (Li et al., 2019). Feeding pregnant rats a high-salt diet can cause changes in the gut microbiota and result in offspring exhibiting ASD-like behaviors (Afroz et al., 2021). The imbalance of gut microbiota in parents of children with autism may be related to genetic susceptibility and lifestyle factors, highlighting the importance of considering parental gut microbiota alongside that of affected children.

This study is the first to systematically compare the multi-omics characteristics of families with ASD (including children and parents) and reveals abnormalities in the microbial-metabolic network at the family level, providing evidence for the “family microbiome transmission” hypothesis of ASD. The mechanisms of microbial transmission mainly involve vertical transmission [such as childbirth (Murphy et al., 2023) and breastfeeding (Zhao et al., 2021)] and horizontal transmission [such as skin contact (Baniel and Charpentier, 2024)]. One study has indicated that the maternal microbiome is crucial for children’s neurodevelopment, particularly the influence of the mother’s gut microbiome on early fetal development (Orchanian and Hsiao, 2025). The main factors affecting microbial transmission at the family level include genetic factors (Griffiths et al., 2019), environmental factors (Leung et al., 2021), and lifestyle (Giacaman, 2018). The gut microbiomes of children with autism are significantly different from those of Non-ASD children, and these differences are closely related to changes in cognitive function and social behavior (Cryan and O’Mahony, 2011). The transmission of the gut microbiota within families may assist in exploring the etiological factors of autism.

Although this study has achieved some results in studying the oral gut microbiomes and metabolites of families with autism compared to Non-ASD families, there are still certain limitations. First, the sample size of this study was relatively small, and there were certain differences in the sex composition of the autism and control groups, as well as age differences between parent groups (*p* = 0.006), which may have introduced confounding factors. Second, this was a cross-sectional study, which had certain limitations in determining causal relationships. At the same time, due to the inclusion of different genders and varying GI manifestations in the population, these factors may lead to certain biases in the results. Finally, the resolution limits of the 16S rRNA sequencing and LC–MS techniques used in this study prevented the in-depth analysis of strain or metabolite functional mechanisms.

However, this study also proposes new directions for future translational applications, such as targeting the oral *Neisseria*, *Caulobacter*, or glutamine pathways for microbial interventions (such as probiotics or dietary adjustments) that may improve ASD symptoms; exploring the relationship between differential gut microbes and SNPs; and investigating new oral gut ASD microbes or metabolites in parents for early intervention in infants and toddlers. Future studies should expand the sample size and conduct longitudinal cohort analyses to verify causal relationships, integrate metagenomic or metabolomic deep sequencing to explore mechanisms, conduct animal model experiments to verify the effects of the microbial-metabolic axis on neurobehavior, and evaluate the clinical effects of oral-gut microbiome family intervention strategies.

## Data Availability

The original contributions presented in the study are publicly available. This data can be found here: 10.6084/m9.figshare.30119203.

## Supplementary material

SUPPLEMENTARY FIGURE S1 Comparison of oral microbiome 16s RNA sequencing data. ASD-C, autism Child; ASD-P, family members of autism child; N-C, Non-ASD child; N-P, family members of Non-ASD child. **(A)** Top 30 relative abundance of genus between four groups. **(B)** Sankey plots of genus abundance between four groups. **(C)** Genus correlation heatmap of Top 30 abundance between four groups. **(D)** Different function prediction with MetaCyc database between ASD-C and ASD-P group.

SUPPLEMENTARY FIGURE S2 16s RNA sequencing data comparison of fecal microbiota. ASD-C, autism Child; ASD-P, family members of autism child; N-C, Non-ASD child; N-P, family members of Non-ASD child. **(A)** Venn diagram of fecal microbial species distribution in each group. **(B)** Chao1 index of Alpha diversity between four groups. **(C)** Shannon index of Alpha diversity between four groups. **(D)** Principal coordinates analysis of groups between four groups.

SUPPLEMENTARY FIGURE S3 Analysis results of differential metabolites in feces. ASD-C, autism child; ASD-P, family members of autism child; N-C, Non-ASD child; N-P, family members of non-ASD child. **(A)** Bar chart of fecal Ala-Gly content differences between ASD-C and N-C. **(B)** Bar chart of fecal Ala-Gly content differences between ASD-C and ASD-P. **(C)** Venn diagram comparing each group pairwise. **(D)** *K*-means clustering analysis chart of differential metabolites in all groups.

SUPPLEMENTARY FIGURE S4 ROC curve of differential metabolites in feces. ASD-C, autism Child; ASD-P, family members of autism child; N-C, Non-ASD child; N-P, family members of Non-ASD child. **(A)** ROC curve of Ala-Gly in ASD-C vs. ASD-P. **(B)** ROC curve of Ala-Gly in ASD-C vs. N-C. **(C)** ROC curve of 2-(Dipentylamino)-2-(hydroxymethyl)-1,3-propanediol in ASD-C vs. N-C. **(D)** ROC curve of 2-(Dipentylamino)-2-(hydroxymethyl)-1,3-propanediol in ASD-P vs. N-P.

SUPPLEMENTARY FIGURE S5 Comparison of oral microbiota 16s RNA between families with autism spectrum disorder (ASD-C+P) and Non-ASD families (N-C+P). **(A)** Comparison of oral microbiota Alpha Diversity between ASD-C+P and N-C+P. **(B)** Bubble Plot comparing the top 30 abundance of oral microbiota at the Phylum level between ASD-C+P and N-C+P. **(C)** Barplot comparing the abundance of oral microbiota at the genus level between ASD-C+P and N-C+P. **(D)** Oral Genus correlation heatmap of differential abundance between ASD-C+P and N-C+P.

SUPPLEMENTARY FIGURE S6 Analysis results of fecal differential metabolites. ASD-C+P, metabolites of feces from families with autism; N-C+P, metabolites of feces from Non-ASD families. **(A)** Heatmap of the top 10 significantly upregulated and downregulated differential substances (ASD-C+P vs. N-C+P). **(B)** Bar chart of significantly downregulated differential metabolites (ASD-C+P vs. N-C+P). **(C)** Bar chart of significantly upregulated differential metabolites (ASD-C+P vs. N-C+P). **(D)** Differential fecal metabolites correlation heatmap between ASD-C+P and N-C+P.
