# Supplementary figures and images for: Parents-child multiple sites of microbial and metabolic signatures in autism spectrum disorder
Source: Front Microbiol. 2026 Jan 22;16:1745874. doi: 10.3389/fmicb.2025.1745874 (PMC12872929; doi:10.3389/fmicb.2025.1745874)

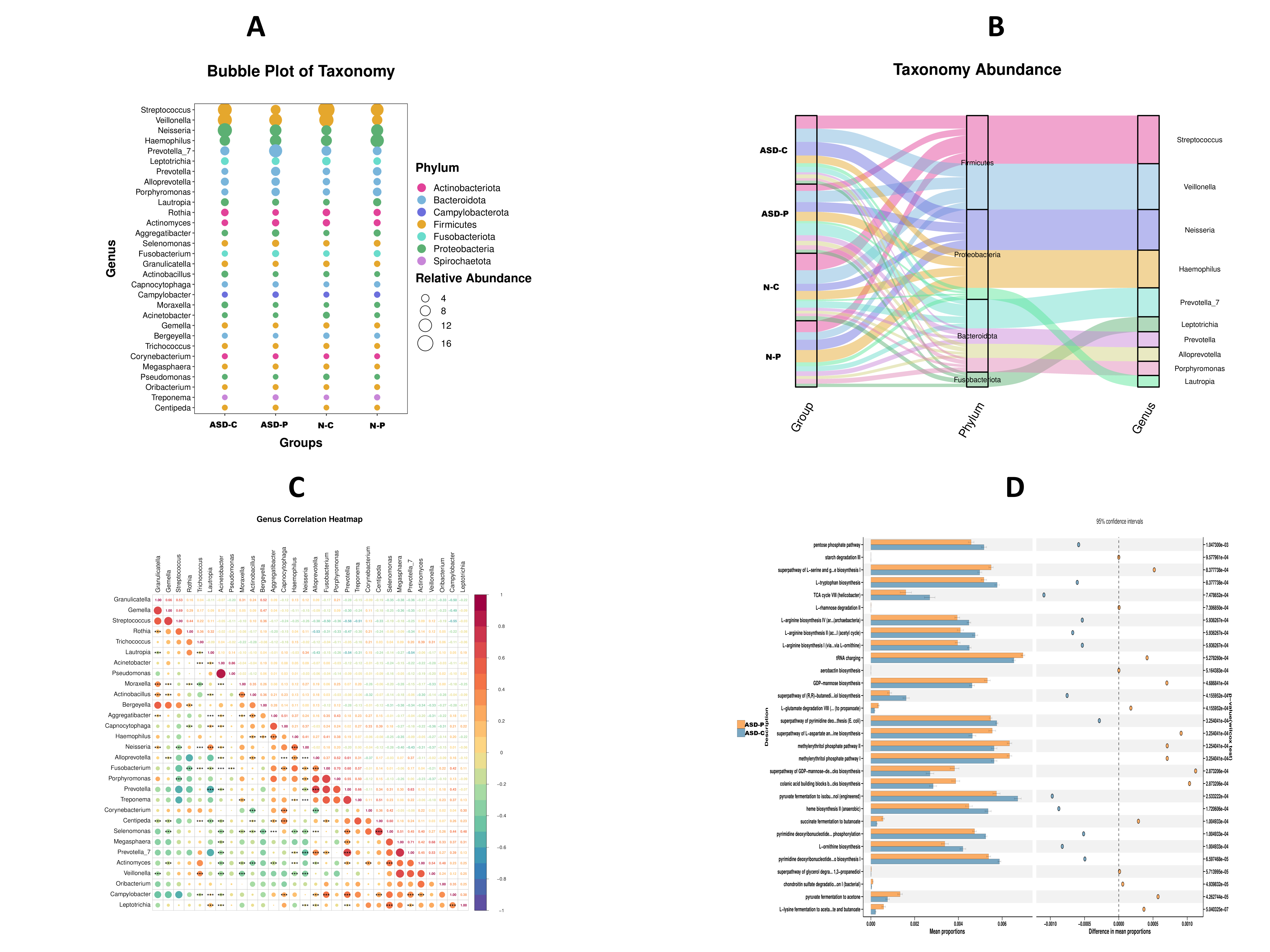

Supplement: SUPPLEMENTARY FIGURE S1 — Comparison of oral microbiome 16s RNA sequencing data. ASD-C, autism Child; ASD-P, family members of autism child; N-C, Non-ASD child; N-P, family members of Non-ASD child. (A) Top 30 relative abundance of genus between four groups. (B) Sankey plots of genus abundance between four groups. (C) Genus correlation heatmap of Top 30 abundance between four groups. (D) Different function prediction with MetaCyc database between ASD-C and ASD-P group. [file Image_1.tif]

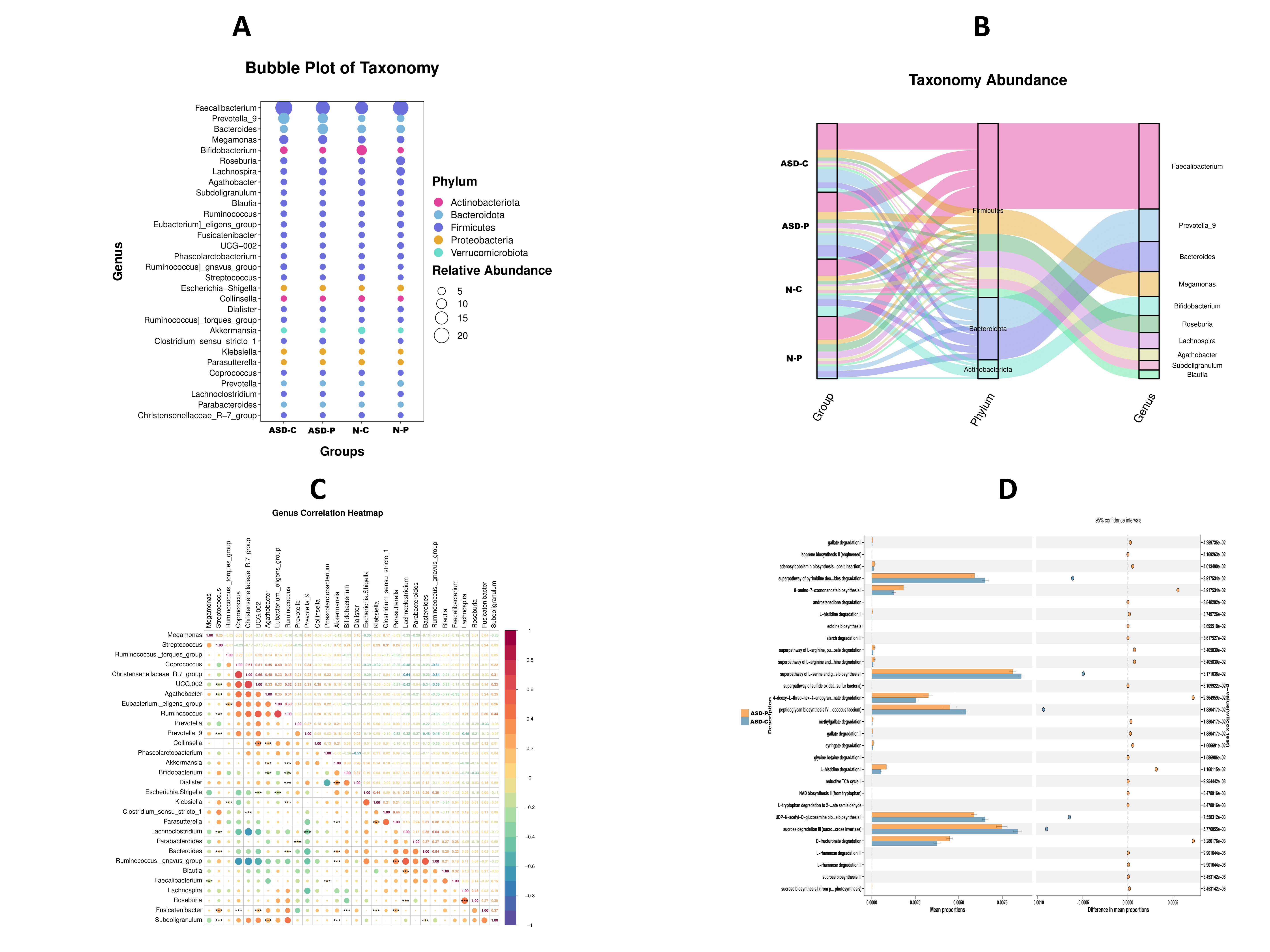

Supplement: SUPPLEMENTARY FIGURE S2 — 16s RNA sequencing data comparison of fecal microbiota. ASD-C, autism Child; ASD-P, family members of autism child; N-C, Non-ASD child; N-P, family members of Non-ASD child. (A) Venn diagram of fecal microbial species distribution in each group. (B) Chao1 index of Alpha diversity between four groups. (C) Shannon index of Alpha diversity between four groups. (D) Principal coordinates analysis of groups between four groups. [file Image_2.tif]

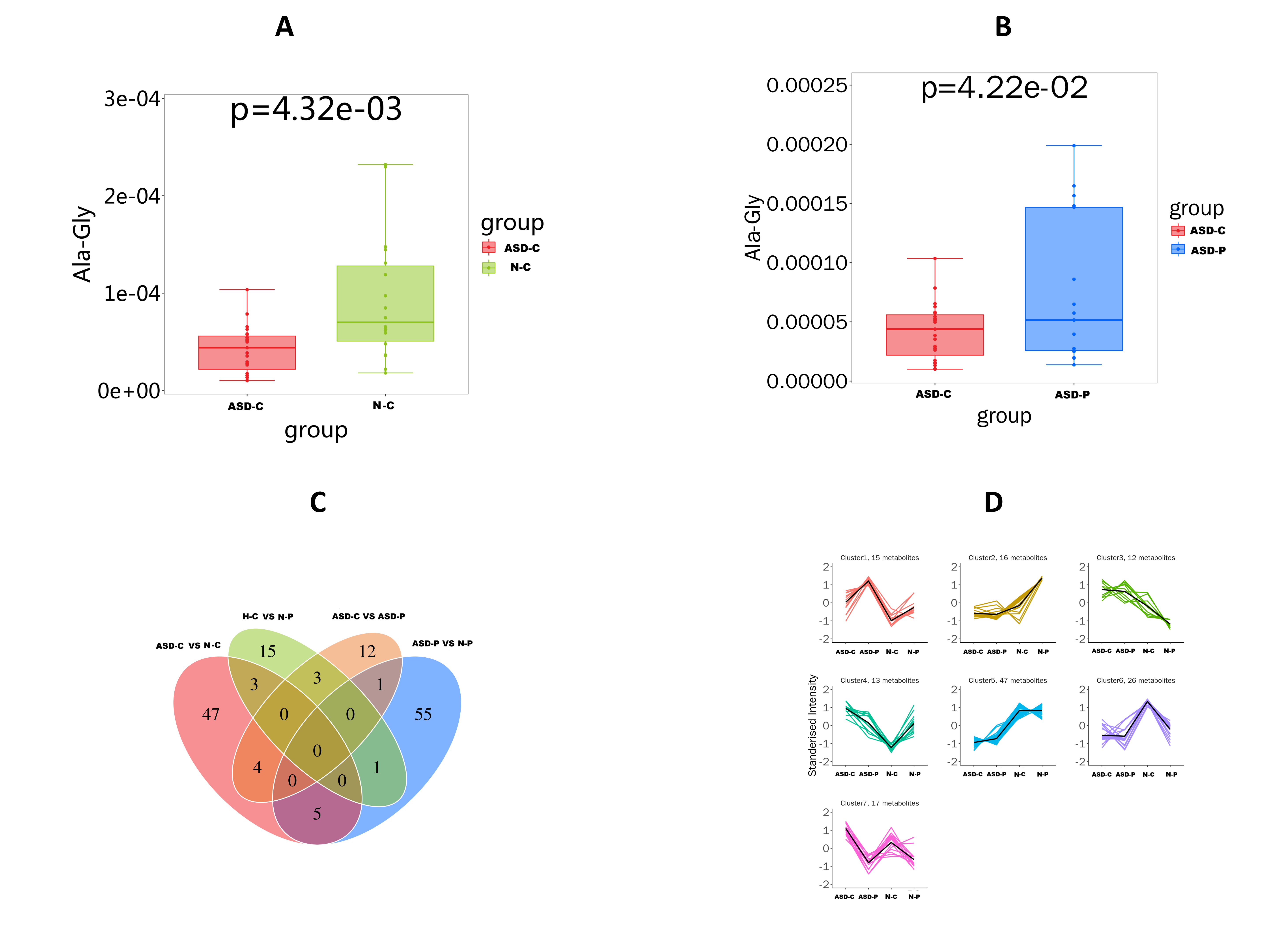

Supplement: SUPPLEMENTARY FIGURE S3 — Analysis results of differential metabolites in feces. ASD-C, autism child; ASD-P, family members of autism child; N-C, Non-ASD child; N-P, family members of non-ASD child. (A) Bar chart of fecal Ala-Gly content differences between ASD-C and N-C. (B) Bar chart of fecal Ala-Gly content differences between ASD-C and ASD-P. (C) Venn diagram comparing each group pairwise. (D) K-means clustering analysis chart of differential metabolites in all groups. [file Image_3.tif]

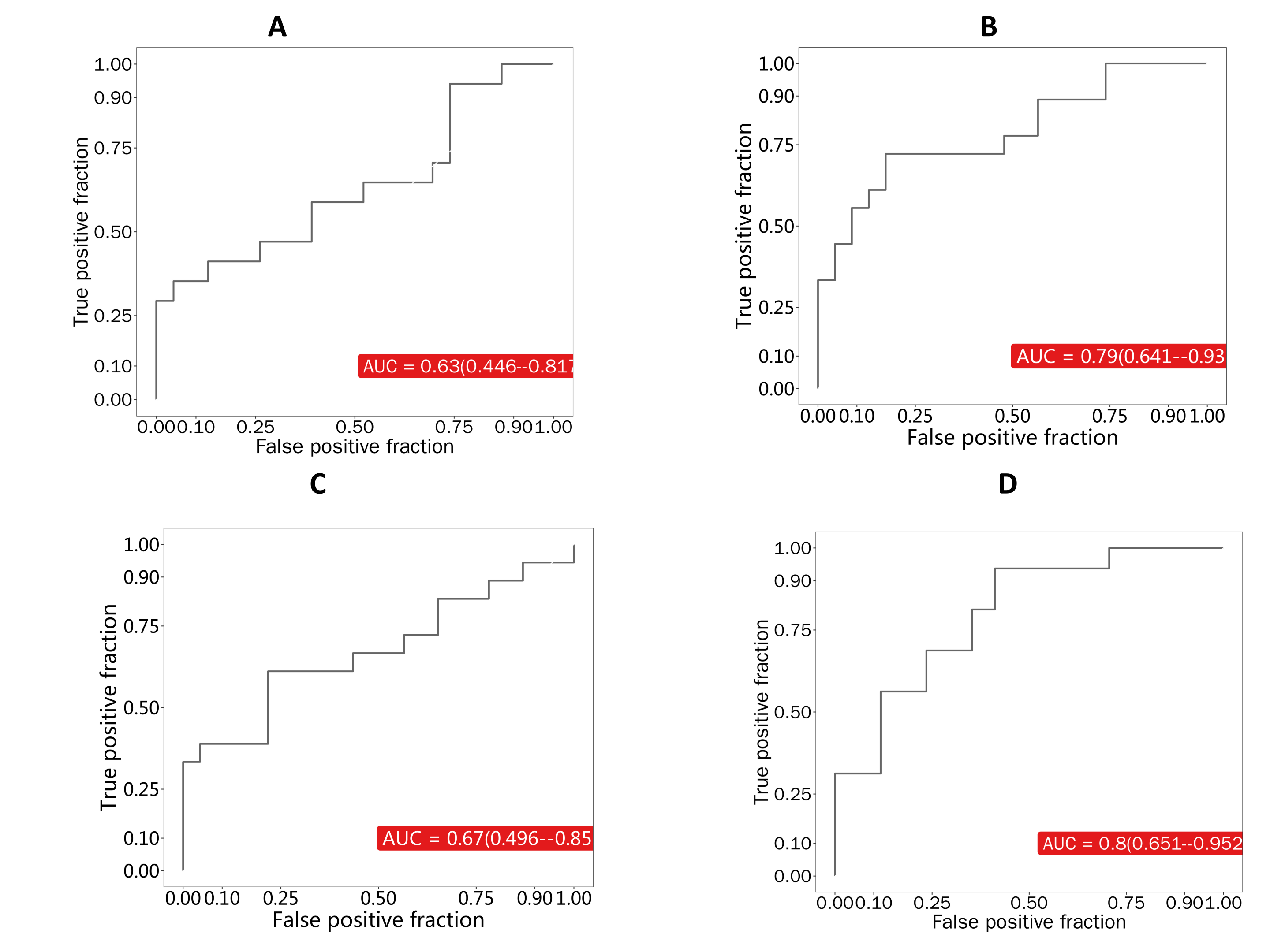

Supplement: SUPPLEMENTARY FIGURE S4 — ROC curve of differential metabolites in feces. ASD-C, autism Child; ASD-P, family members of autism child; N-C, Non-ASD child; N-P, family members of Non-ASD child. (A) ROC curve of Ala-Gly in ASD-C vs. ASD-P. (B) ROC curve of Ala-Gly in ASD-C vs. N-C. (C) ROC curve of 2-(Dipentylamino)-2-(hydroxymethyl)-1,3-propanediol in ASD-C vs. N-C. (D) ROC curve of 2-(Dipentylamino)-2-(hydroxymethyl)-1,3-propanediol in ASD-P vs. N-P. [file Image_4.tiff]

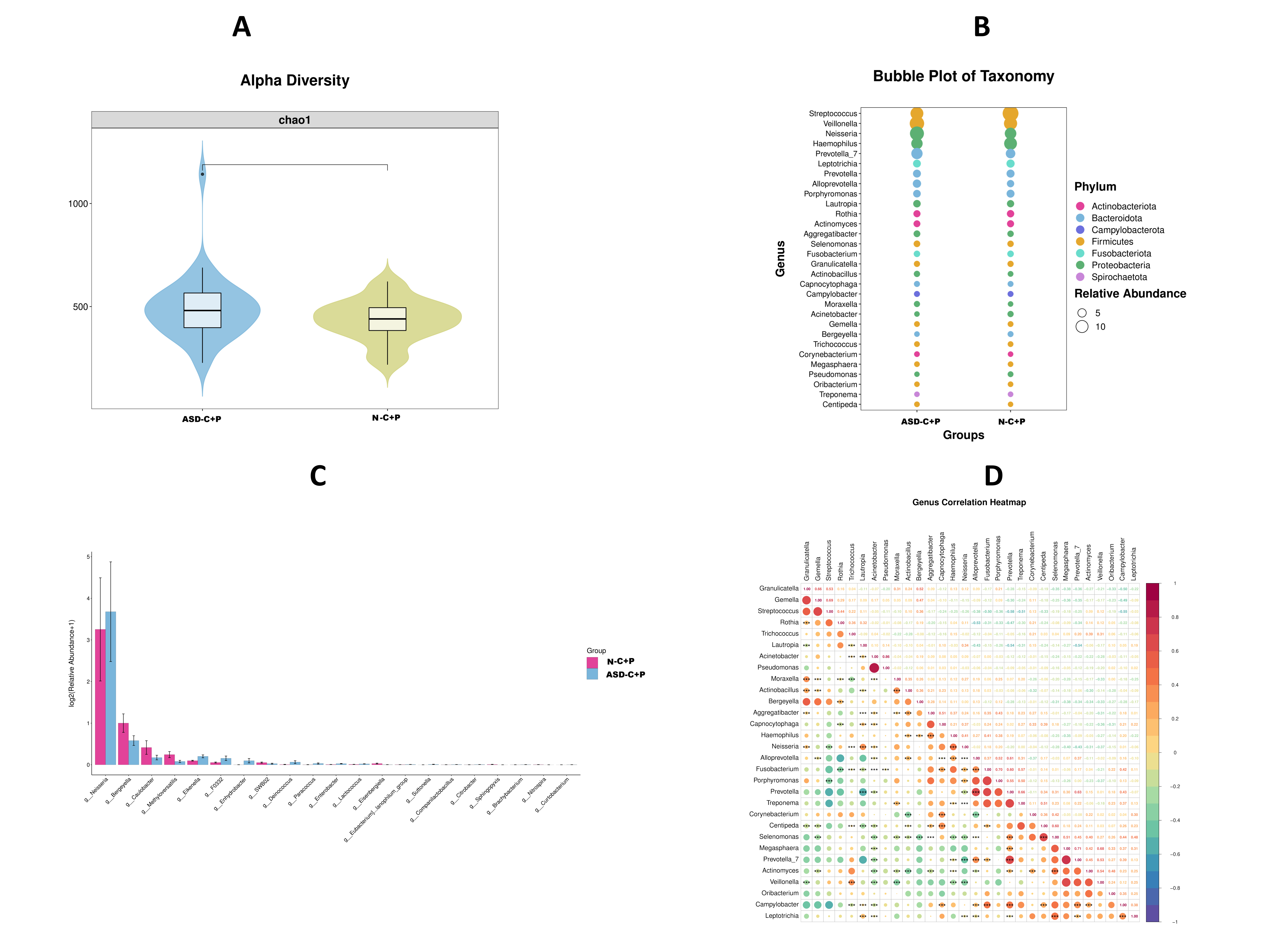

Supplement: SUPPLEMENTARY FIGURE S5 — Comparison of oral microbiota 16s RNA between families with autism spectrum disorder (ASD-C+P) and Non-ASD families (N-C+P). (A) Comparison of oral microbiota Alpha Diversity between ASD-C+P and N-C+P. (B) Bubble Plot comparing the top 30 abundance of oral microbiota at the Phylum level between ASD-C+P and N-C+P. (C) Barplot comparing the abundance of oral microbiota at the genus level between ASD-C+P and N-C+P. (D) Oral Genus correlation heatmap of differential abundance between ASD-C+P and N-C+P. [file Image_5.tif]

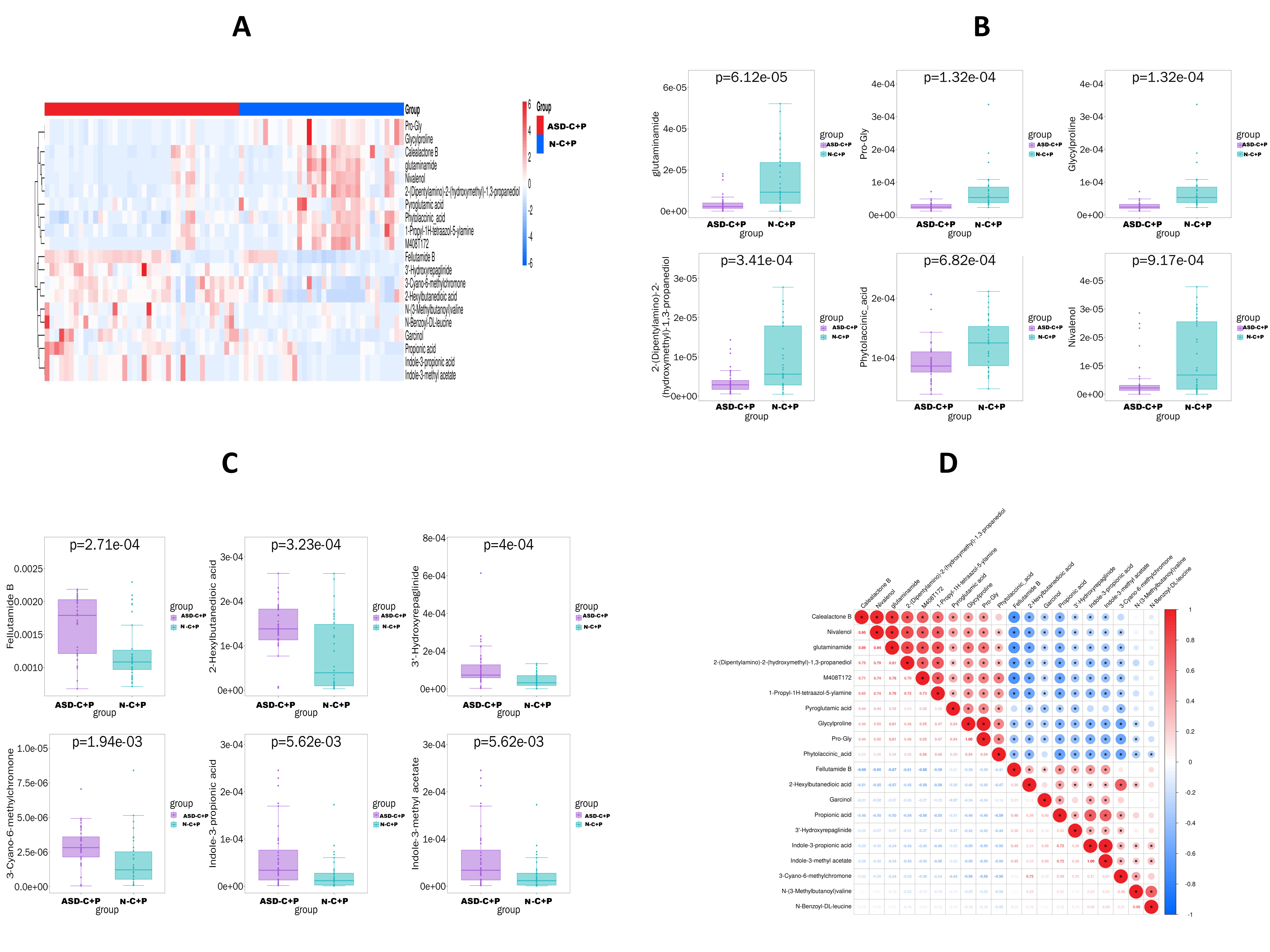

Supplement: SUPPLEMENTARY FIGURE S6 — Analysis results of fecal differential metabolites. ASD-C+P, metabolites of feces from families with autism; N-C+P, metabolites of feces from Non-ASD families. (A) Heatmap of the top 10 significantly upregulated and downregulated differential substances (ASD-C+P vs. N-C+P). (B) Bar chart of significantly downregulated differential metabolites (ASD-C+P vs. N-C+P). (C) Bar chart of significantly upregulated differential metabolites (ASD-C+P vs. N-C+P). (D) Differential fecal metabolites correlation heatmap between ASD-C+P and N-C+P. [file Image_6.tif]

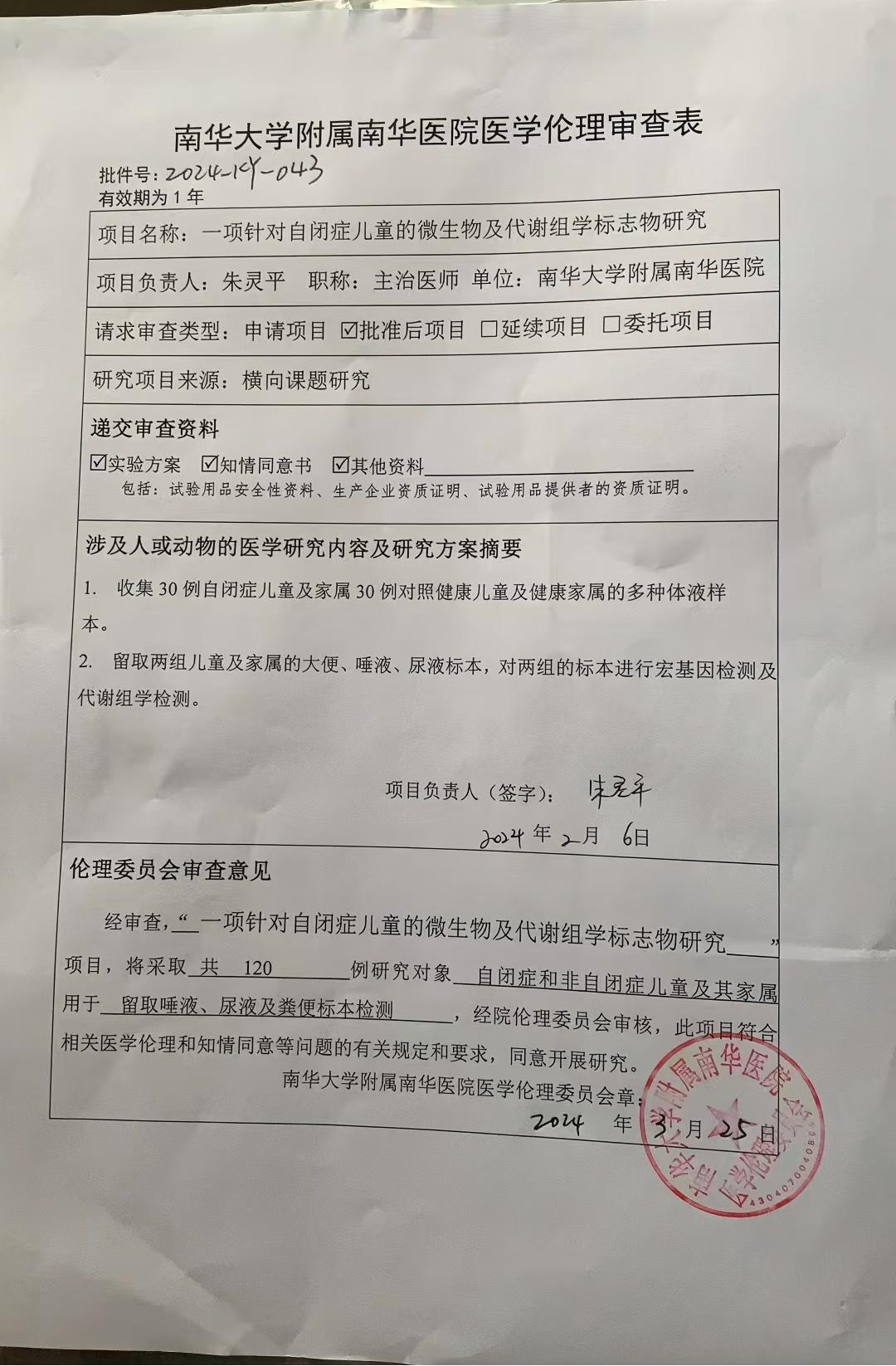

Supplement: Supplementary file 7 [file Image_7.jpeg]
